# Intersectional Effects of Crystal Features on the Actuation Performance of Dynamic Molecular Crystals

**DOI:** 10.1021/jacs.3c02184

**Published:** 2023-05-26

**Authors:** Jad Mahmoud Halabi, Marieh B. Al-Handawi, Rodrigo Ceballos, Panče Naumov

**Affiliations:** † Smart Materials Lab, New York University Abu Dhabi, P.O. Box 129188, Abu Dhabi, UAE; ‡ Paige.ai, New York City, New York 10036, United States; § Center for Smart Engineering Materials, New York University Abu Dhabi, P.O. Box 129188, Abu Dhabi, UAE; ∥ Research Center for Environment and Materials, Macedonian Academy of Sciences and Arts, Bul. Krste Misirkov 2, Skopje MK−1000, Macedonia; ⊥ Molecular Design Institute, Department of Chemistry, New York University, 100 Washington Square East, New York, New York 10003, United States

## Abstract

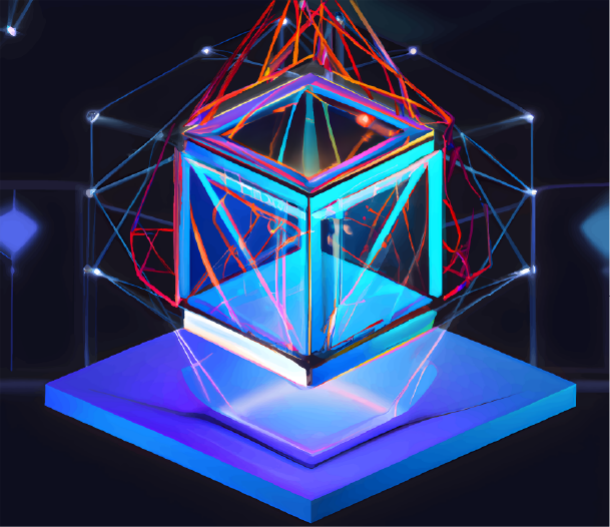

Despite being researched for decades, shape-shifting molecular crystals have yet to claim their spot as an actuating materials class among the primary functional materials. While the process for developing and commercializing materials can be lengthy, it inevitably starts with building an extensive knowledge base, which for molecular crystal actuators remains scattered and disjointed. Using machine learning for the first time, we identify inherent features and structure–function relationships that fundamentally impact the mechanical response of molecular crystal actuators. Our model can factor in different crystal properties in tandem and decipher their intersectional and combined effects on each actuation performance. This analysis is an open invitation to utilize interdisciplinary expertise in translating the current basic research on molecular crystal actuators into technology-based development that promotes large-scale experimentation and prototyping.

## Introduction

Knowledge can be public yet remain fragmented and undiscovered if the separately designed information is logically connected but is not retrieved and assembled to disclose patterns unintended and unseen before.^1^ Responsive molecular crystals are a prime example of a class of materials whose application remains rudimentary despite the wealth of reported crystal structures and other physicochemical properties. The short- and long-range intermolecular interactions are known to define both crystallinity and versatile mechanical properties of molecular crystals, govern their energy transduction, and maintain their long-range lattice order despite large deformations.^2,3^ However, the study of mechanical responsive properties of molecular crystals, for the most part, has remained limited to isolated case studies, entirely distracted from the holistic class of actuating materials they represent. With the increased reporting, new application prospects have emerged and commanded the consideration of molecular single crystals as energy-harvesting and work-generating materials.^4−6^ Being at a unique intersection between soft and hard materials,^7^ these crystals appear to be a natural choice for actuation elements in soft and microrobotics.^8^ Nonetheless, a clear definition of the capacity and limitations of molecular crystals as an actuating class is still lacking and currently hinders their incorporation in actuator design. Indeed, many reports on responsive molecular crystals are phenomenological and often fail to thoroughly evaluate their static and dynamic actuation performance. While characterizing the *static* response reveals how these crystals perform in either two states (fully loaded or entirely free), the *dynamic* response is the aspect that evaluates the efficiency of energy transduction into mechanical work under dynamic loading.^9^ Understanding the structure–function relationships through mapping out the working principles of each crystal example could explain the behavior of the whole materials class. It would facilitate discovering new exceptional dynamic molecular crystals and enable controlling, optimizing, and possibly customizing the performance of previously studied crystals. While some solid-state molecular transformations that drive the mechanical response of molecular crystals have been considered dramatic, these characteristics do not necessarily translate into impressive actuation behaviors. Considering the chemical makeup in isolation of mechanical properties and crystal morphologies will often fall short in explaining the actuation performance. Photodimerization reactions in molecular crystals, for example, can result in crystal bending, axial expansion/contraction, or even twisting,^10−15^ leading to a wide range of actuation performance. Without understanding the intersectional effects of various chemical and physical features on the actuation performance, much will likely remain undiscovered about this emerging family of engineering materials.

Ensemble learning methods have been implemented to guide the search for hard materials and predict the mechanical properties of those not yet characterized.^16^ However, no similar efforts have been employed yet to explore the vast number of reported molecular crystals. Collating a complete and extensive dataset remains the biggest challenge in inviting exploration of these materials through machine learning methods and accurate prediction models. While there have been attempts to create a dataset of the mechanical properties of organic crystals, the analysis remained statistical.^7^

In this work, the first comprehensive dataset of over 100 mechanically responsive molecular crystals reported and characterized to date was compiled (see Data S1 and Supporting Experimental Methods, Supporting Information). All possible actuation performance indices were collected directly, calculated, or extracted from the data, images, and videos provided in the reports along with common categorical features, X-ray diffraction information, and chemical, physical, and mechanical properties (see Tables S1–S4, Supporting Information). A statistical analysis was conducted followed by establishing a machine learning model, for the first time, that reveals the interconnected patterns and relationships between different crystal features and their collective impact on each actuation performance index. Here, we aim to initiate a continuing holistic study, highlight critical data gaps needed to prepare the field for fully utilizing machine learning tools, and invite interdisciplinary collaborations in order to fast-track the advancement of molecular crystals to the forefront of practical applications.

## Results and Discussion

**Figure 1:**
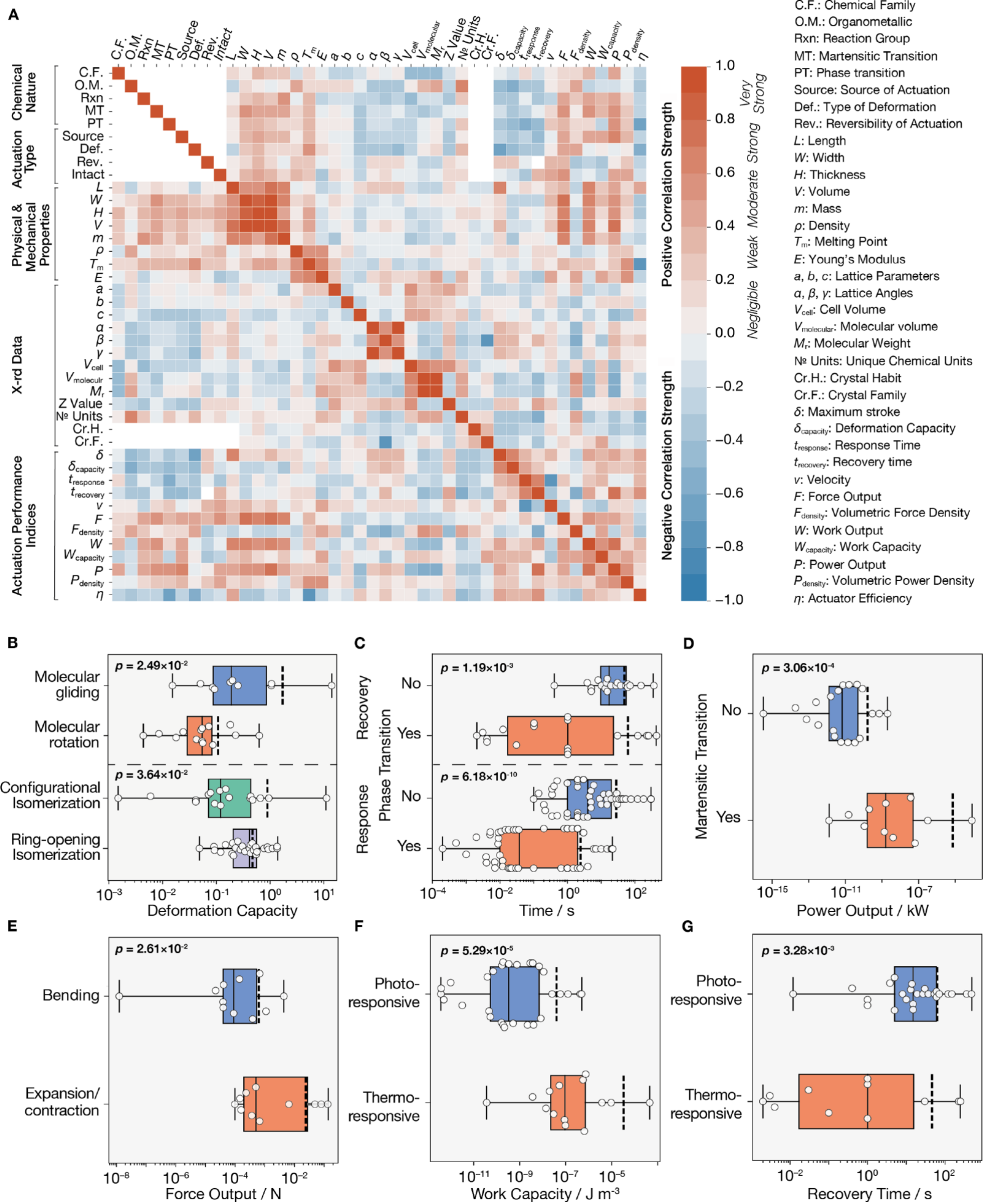
Statistical analysis of the performance of dynamic molecular crystals. (A–G) Correlation matrix of all crystal properties considered (A), and two-sample *t*-tests showing significant distinctions (with *p*-values reported) between pairwise combinations of categories with respect to performance indices (B–G). The effect of the type of chemical reaction on the deformation capacity (B), recovery/response time (C), and the power output (D). (E) The difference in force output between bending and expanding/contracting crystals. (F,G) The difference in work output and recovery time between photoresponsive and thermoresponsive crystals. The data is plotted on the log scale. The vertical dashed lines represent the mean. Outliers were not determined, and the minimum and maximum include the full range of the data.

In order to highlight important associations between crystal features and actuation performance indices, a Spearman correlation matrix that measures any monotonic pairwise relationship between all variables considered in this study was constructed (Figure 1A). The Spearman’s rank correlation coefficient was later used to cross-check feature importance in the prediction model and rule out redundancies and negligible associations. Two-sample *t*-tests were conducted to further validate positive and negative categorical correlations between different crystal subgroups and their actuation performance (see Table S5, Supporting Information). All crystal categories determined to have a significant pairwise difference in performance (*n* ≥ 10, *p*-value <0.05, and corresponding confidence interval of 95%) are considered features of fundamental relevance to the actuation behavior of molecular crystals. While crystals responsive to any stimuli were considered, photo- and thermo-activated molecular crystals were found to be the primary contributors to the reported examples of this materials class. Photoisomerization reactions in azobenzenes and hydrazides,^17−21^ [2 + 2] and [4 + 4] photodimerization in anthracenes and organic acids,^12,22−24^ and cyclization of diaryelethenes^25−27^ are some of the most commonly reported photoinduced molecular transformations in organic single crystals. Thermoresponsive crystals can undergo a variety of molecular transformations that can be categorized under conformational changes, molecular gliding, molecular rotation, or a combination thereof.^28^

Figure 1B–G highlights some of the important trends determined by the statistical analysis and validates preliminary generalizations that can be made regarding the actuation performance of specific subgroups of molecular crystals. For instance, molecular gliding appears to allow for higher deformation capacity than molecular rotation. Here, we define deformation capacity as the free stroke per unit length along the axis of actuation. Molecular rotation is sterically more constrained than gliding, which translates into smaller deformations (Figure 1B). Ring-opening isomerization reactions are also more conducive to higher deformation compared to configurational isomerization reactions for the same reason. Crystals that undergo phase transitions or martensitic phase transitions have a considerably different actuation performance in terms of response time, recovery time, and power output compared to those that do not (Figure 1C,D). This can be explained by the rapid nature of these transformations and their direct effect on the mechanical response.

The molecular transformations driving the actuation also determine the amount of induced strain in the single crystal, its directionality, and whether it is a surface phenomenon or a single-crystal-to-single-crystal transformation. When strain is gradient-like and only accumulates on the surface of the crystal, the deformation occurs as bending or twisting.^29,30^ Diffusion-free strains induced homogenously throughout the crystal structure produce anisotropic expansions and contractions.^28,31,32^ Depending on the kinetics and intensity of the chemical reaction and the mode of dissipation of the resulting elastic energy, the response might be in the form of motility, such as jumping, or even disintegration, costing the crystal its structural integrity and causing it to shatter.^33−35^ Given the different mechanics involved, the type of deformation inevitably has a direct effect on the amount of force that molecular crystals can generate (Figure 1E). Finally, comparing thermoresponsive and photoresponsive crystals indicates that the former produce more mechanical work and recover faster (Figure 1F,G). Thermoresponsive crystals’ recovery is dependent on the rate of heat dissipation since most heat-driven mechanical responses are due to abrupt phase transitions that are reversed as soon as a trigger temperature is reached. Photoresponsive crystals are often limited by the rate of the reverse reaction, which in many cases are rather slow reactions and need additional stimuli to accelerate them.

**Figure 2:**
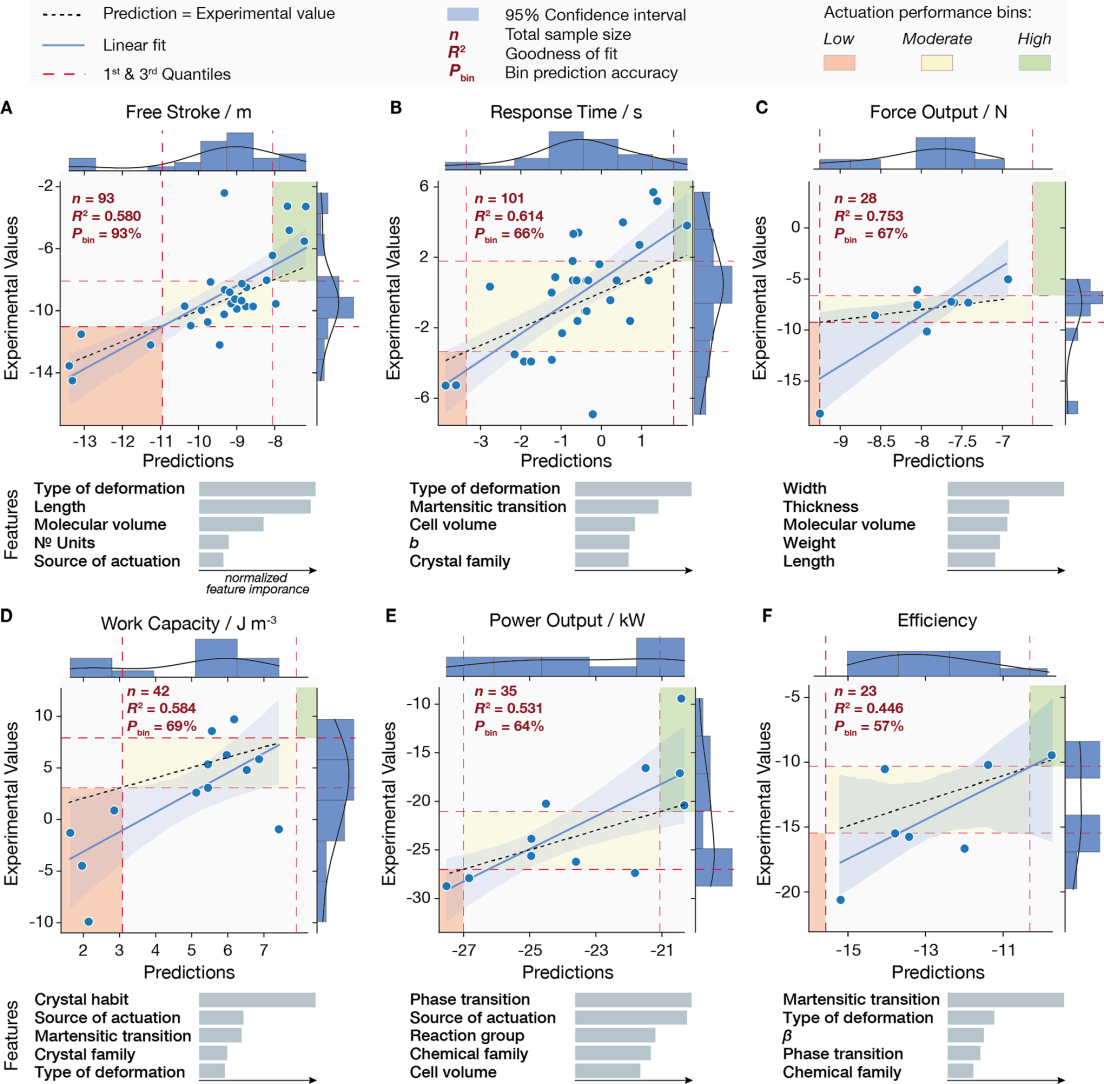
Gradient boosting regression. (A–F) Model predictions on the natural log scale for stroke (A), response time (B), force output (C), work capacity (D), power output (E), and efficiency (F). *R*^2^ represents the goodness of fit of predicted and experimental observations, and *P*_bin_ is the model’s accuracy in binning the performance values into three ranges of low, moderate, and high actuation performance. The solid line represents the linear regression of the predicted values, and the dotted line represents the boundary where the prediction values match the experimental results. The red dashed lines are the first and third quartiles, Q1 and Q3. The blue-shaded area represents the 95% confidence interval for each regression. For each actuation performance index, the top-ranking features with the five highest feature importance scores are listed in order.

Responsive molecular crystals can exhibit wide combinations of properties that prove to have a complex influence on the actuation performance beyond the direct effect that each crystal feature might have independently. Certain features might be overshadowed by others, and their effect can either be amplified or diminished, which further complicates drawing direct conclusions. Supported by the statistical analysis, ensemble learning methods were employed to understand the interconnectedness of crystal features. To explore potential patterns in the performance of each crystal in relation to its entire chemical makeup and physical properties, we constructed a machine learning model through a gradient boosting algorithm that accounts for categorical features. Gradient boosting operates by sequentially combining multiple learning models and is suitable for the sample size at hand.^36,37^ Each model attempts to minimize the prediction error by learning from its predecessor’s. The final ensemble model, a weighted sum of all previous models, estimates the importance of each crystal feature in predicting the performance indices. We split the dataset into training and testing subgroups with an optimized ratio of 7:3, respectively. After training the model, the testing data was used to validate the model’s predictions across eight different actuation metrics. The tree depth, learning rate, and regularization parameters were tuned to optimize the model, and the training and validation curves were monitored to detect anomalies and prevent overfitting (see Figure S1, Supporting Information). The most critical predictions are shown in Figure 2. *R*^2^ was calculated to determine how much of the variation in the dependent features (actuation performance metrics) can be explained by the independent crystal features used as descriptors (Figure 2A–F); therefore, *R*^2^ is treated as the statistical significance of the explanatory power rather than the prediction accuracy of the model.

Given the inherent trade-off between the interpretability and accuracy of any prediction model and since our focus lies in understanding the relationship between crystal features and the actuation performance, a parametric method was used to estimate the latter. Such method entails simplifying the prediction of the actuation performance through a first-order parametric form of crystal features and ranking them according to feature importance scores that reflect their importance in predicting any actuation performance metric (see Table S6, Supporting Information). Due to the lack of measurement errors in the literature, all data points were treated as true values with equal weights and the dataset could not be tested for outliers. Missing data, particularly for neglected performance metrics or ones that are experimentally complicated to determine, such as work, power, and efficiency, also contributed to lower prediction accuracies. Nonetheless, the model can successfully determine the crystal features of primary influence on each actuation performance metric. Here, the model’s explanatory power of the associations made was prioritized over prediction accuracy, which could be drastically improved by exploring higher-order parameters and resorting to a black-box approach. However, the latter approach renders the crystals’ structure–function associations practically uninterpretable, which defeats the purpose of this study. The predictions were also binned into low, moderate, and high actuation performance ranges determined by the quantiles of the entire dataset. Hence, this model can be used to focus on finding new responsive molecular crystals with high actuation capacity as a first line of screening. Predictions for the range of free stroke have the highest accuracy with 93%. The prediction accuracy for the remaining actuation performance metrics can be drastically improved by more extensive reporting on these properties. The associations between each actuation property and its corresponding crystal features on which the machine learning model relies on heavily are dissected next.

### Free Stroke

In predicting the maximum free stroke of responsive molecular crystals, the model appears to depend heavily on the type of deformation, crystal length, molecular volume, number of unique chemical units, and source of actuation. Bending crystals often exhibit large strokes since the mechanical deformations are not directly proportional to the induced crystal lattice strains, unlike axial crystal expansions and contractions. Given the narrow range of lattice strain exhibited in responsive molecular crystals,^38^ the crystal length, which is often along the axis of actuation, naturally plays an important role in determining the free stroke. Interestingly, single crystals with smaller molecular volumes and lower unique chemical units per unit lattice appear to have larger free strokes. Smaller and simpler molecules are associated with less energy-demanding molecular transitions and reconfigurations, which are more conducive to an amplified actuation strain and larger strokes. Additionally, thermoresponsive molecular crystals appear to produce a distinctive range of free stroke compared to photoresponsive crystals, making the source of actuation a critical feature.

### Response Time

The crystal features with the highest feature importance score for determining the response time are the type of deformation, martensitic transitions, unit cell volume, *b* lattice dimension, and crystal family. Different types of deformation occupy distinctive ranges in response time, with bending crystals, on average, being the slowest. Given their homogenous self-sustaining phase transitions, martensitic molecular crystals can also be distinguished from non-martensitic crystals when compared by their response time, which explains the model’s reliance on this feature. Unit cell dimensions are also important features, possibly due to their effect on the density of molecular packing, which influences energy transfer within the structure and, subsequently, the response time.

### Force Output

The force output of responsive molecular crystals is heavily influenced by the crystal’s physical properties. The dimensions and weight of the crystals are directly proportional to their force output despite changes in other crystal features. Higher crystal width and thickness contribute to a larger cross-sectional area and, consequently, a larger force resistance. The free stroke or displacement is directly proportional to crystal length, which explains the latter’s subsequent effect on force generation. However, additional data on the mechanical properties of molecular crystals is essential to factor in the inversely proportional relationship between the length and mechanical stiffness of a crystal and, thereby, its capacity to generate force. The molecular volume also influences the force output since single crystals with smaller molecules have some of the highest force outputs reported.

### Work Capacity

When it comes to predicting the work capacity of molecular crystals, the crystal habit and crystal family, source of actuation, martensitic transitions, and the type of deformation are all features of high importance. Crystals with high surface-area-to-volume ratios, such as plate-like or tabular crystals, have more surface area exposed to the actuation stimuli and slender structures that facilitate stress dissipation, reduce crack formation, and limit energy loss. This effect explains the reliance of the model on crystal habit and crystal family features to predict the work capacity. The stress distribution in an actuated crystal is also dependent on the type of deformation. In bending crystals, large stresses accumulate at the clamped base of the crystal, mainly when the free end exerts a force against an object. These effects directly impact the energy transduction and, consequently, the mechanical work output. Again, more extensive Young’s modulus data is necessary to understand the impact of molecular crystals’ moderate hardness on the actuation displacement and force output, both of which directly influence the work generation capacity.

Differences between the photo- and thermo-chemistry driving the actuation, particularly in how the input energy is administered, absorbed, and propagated through the crystal structure, also impact the energy transduction into mechanical work. Martensitic phase transitions create homogenous and diffusion-free molecular transformations. As soon as the new phase forms, the energy necessary to drive further transformation is reduced since the crystal structure is stabilized, which translates into stable energy transduction and places martensitic crystals within a distinctive work output range.

### Power Output

The power output of molecular crystals is more evenly influenced by five features: phase transition reactions, the source of actuation, reaction groups, chemical family, and lattice unit cell volume. Crystals that undergo phase transition exhibit rapid molecular transformations that often translate to fast response times at the macro scale and result in a higher power output. Thermoresponsive crystals, particularly those undergoing conformational molecular transformations and molecular gliding, have higher power output than those undergoing configurational transformations. Different chemical families appear to have distinctive ranges of power outputs where the highest values reported are those of organometallic compounds. Generally, the unit cell volume of the crystal lattice is inversely proportional to the power output, possibly due to the tighter packing in crystals with small lattice units.

### Efficiency

Contrary to the common belief that diffusion-free martensitic phase transitions contribute to higher energy conversion efficiency, crystals that undergo phase transitions generally have lower efficiency. While the power output of martensitic crystals can be higher than other crystals due to the unique homogenous nature of these transitions, the high input power required to trigger such transitions has rarely been considered. Given the small sample size of martensitic crystals whose efficiency was retrieved compared to non-martensitic crystals, a more extensive investigation of the efficiency of martensitic transformations is needed to confirm this finding further. Similarly, despite organometallic crystals having higher power output, they appear to require higher power inputs, which detracts from their efficiency. The type of deformation also has an important influence on the efficiency where jumping crystals appear to have low efficiency, likely due to erratic motions that lead to energy dissipation compared to continuous bending that often results from a steady strain buildup. Generally, better reporting on the efficiency of molecular crystal actuators is instrumental, particularly in reporting experimental methodology details. Additionally, the specific heat capacity of thermoresponsive molecular crystals is essential for efficiency calculations, yet it remains underreported.

**Figure 3:**
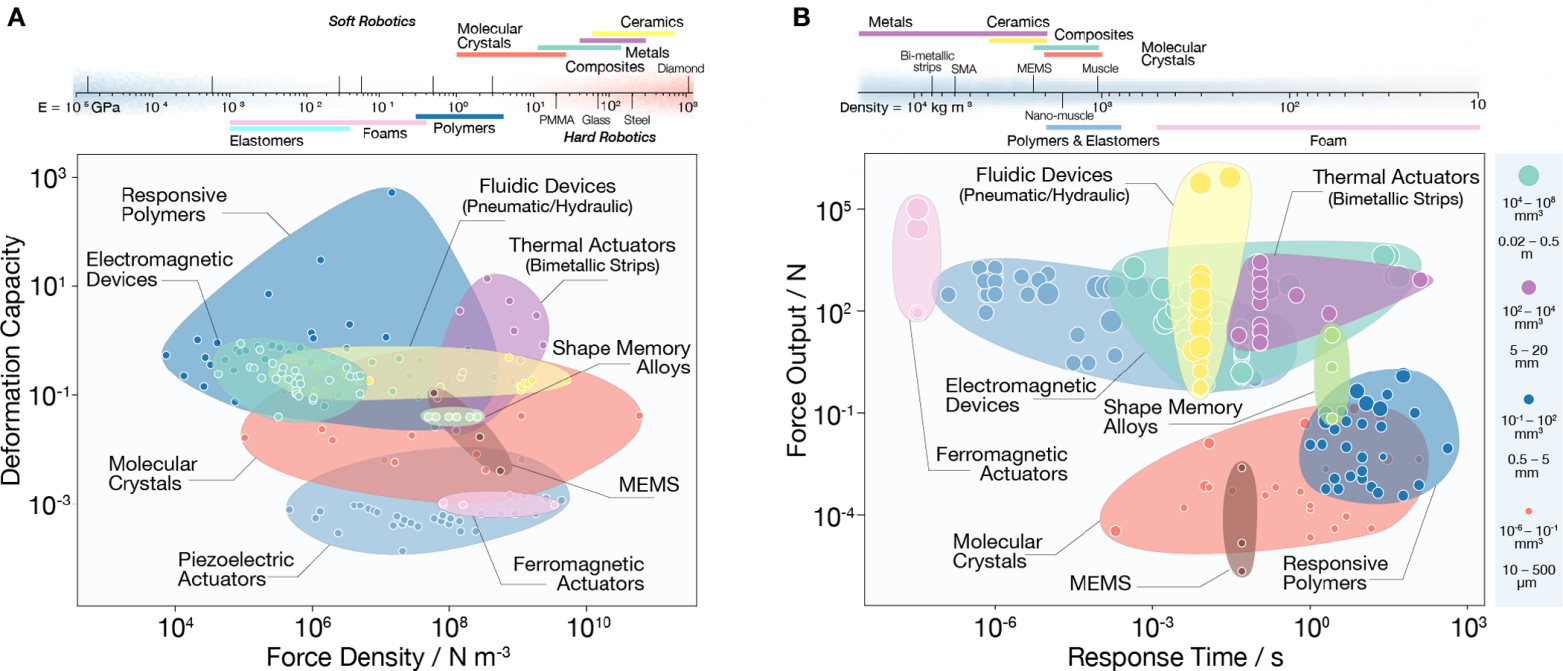
Static performance indices. (A) Actuator deformation capacity versus force density. (B) Response time versus force output for molecular crystals co-plotted with the same attributes for the main actuator classes with a range of soft and hard materials and devices. The translucent envelopes group materials that belong to the same actuator class. In (B), the size of the markers is scaled to reflect the size range of the material.

**Figure 4:**
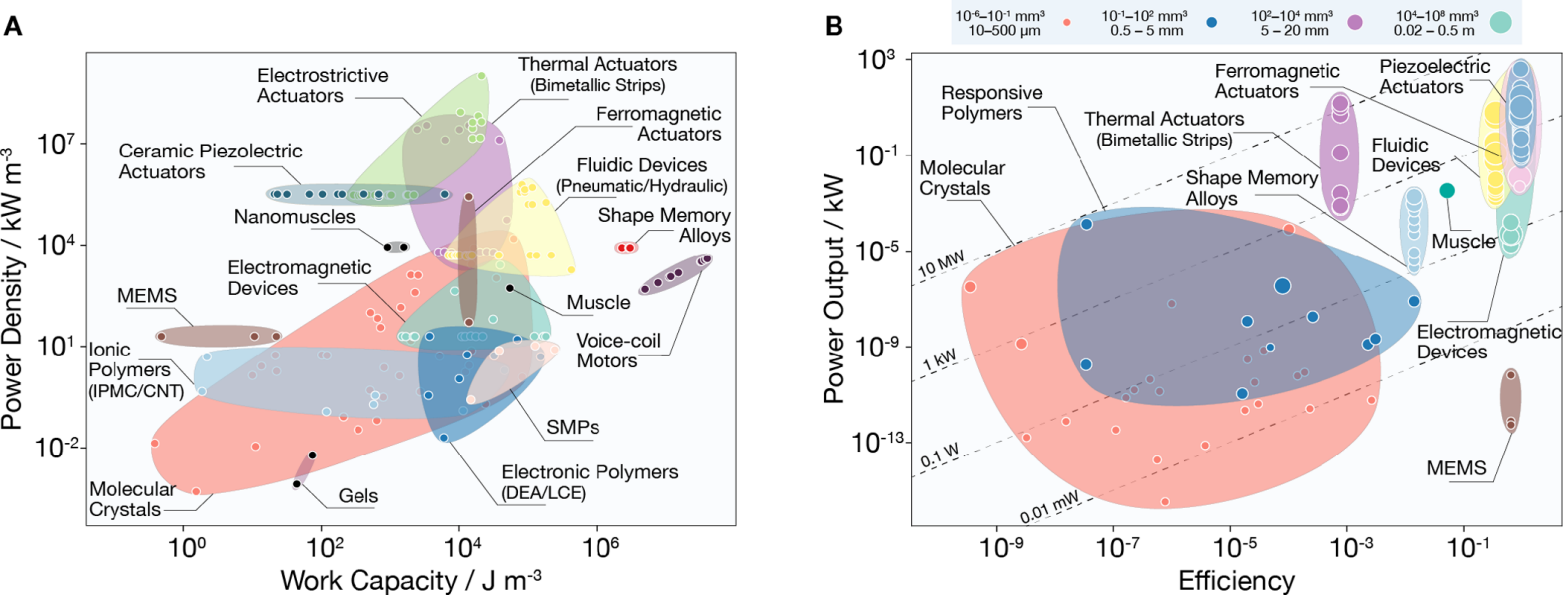
Dynamic performance indices. (A) Actuator power density versus work density. (B) Actuator power density versus energy efficiency for molecular crystals co-plotted with the same attributes for the main actuator classes with a range of soft and hard materials and devices. The translucent envelopes group materials that belong to the same actuator class. In (B), the contour lines link together classes of actuators that operate on the same input power, and the size of the markers is scaled to reflect the size range of the material.

In order to place molecular crystals on the global actuator landscape, Figures 3 and 4 map out the boundaries of performance of molecular crystals to highlight their strengths and weaknesses compared to other actuators (see Supporting Experimental Methods and Table S7, Supporting Information) and their competitive edge for specific applications. As expected, soft materials such as polymers show high deformation capacity but generally have low force generation (Figure 3A). The opposite is true for hard materials such as metals and ceramics. Having moderate Young’s moduli (84% have moduli 1–25 GPa), molecular crystals fill a gap between stiff, high-stress–low-strain actuators and compliant, low-stress–high-strain actuators. They maximize the generated volumetric force density and maintain significant deformation capacity. Compared to soft actuators, their long-range order and higher Young’s modulus contribute to the rapid energy flow within their structures and shorten their response time. We note that molecular crystals have the fastest response time in their size range. The size difference between the actuators is put into context in Figure 3B. Despite being lightweight, molecular crystals can generate high forces up to 0.1 N and at speeds that are 10–1000 times faster than other microactuators. Their performance matches that of gripping and positioning actuators such as micropiezoelectric drivers and electromagnetic motors.^39^ However, unlike rigid actuators, elastic and soft organic crystals can absorb and dissipate undesired excess force generated at the actuator–load interface. This dampening effect turns these materials into better candidates for minimally invasive applications such as cell manipulation and micro-optics.

The global materials property plots also revealed that molecular crystals produce volumetric work and power densities as low as those of gels or surpass high-dynamics actuators like muscles, bimetallic strips, and fluidic devices. The balance between large actuation strain and high output stress—typically mutually exclusive properties—positions molecular crystals as high-work-density actuators. Their volumetric power densities are higher than other soft single-material actuators and comparable to pneumatic devices. Figure 4B shows molecular crystals with power outputs comparable to larger soft polymers and responsive metals and alloys. Despite efficiencies lower than other actuators, most molecular crystals operate at low power inputs ranging between 0.01 and 100 mW. This range makes them safe to operate around biological tissue and diminishes the importance of high efficiency since microactuators require only moderate input powers to generate the small output forces needed.

## Conclusions

As new examples of responsive molecular crystals are unearthed, it is essential to follow standardized performance evaluation methods to avoid undervaluing or overestimating the actuation potential of these unique materials. We strongly recommend that future reports on new crystals include a thorough actuation performance analysis. Physical and mechanical properties such as the Young’s modulus and density are essential to draw better structure–function correlations. Highlighting crystal features of particular importance can guide and accelerate the quest for high-performing molecular crystal actuators, a search that is otherwise short-sighted. Our model can serve as the first level of screening to identify molecular crystals with high actuation capacities prior to any experimental analysis. It is important to reiterate that while some correlations will inevitably appear intuitive given the viable classical mechanics and chemistry logic that explains them, this model serves to determine their importance in dictating the final actuation performance over other features. As we continue to improve this dataset and add new crystal features such as mechanical property measurements, we expect this model to further highlight the interplay between different chemical, physical, and mechanical crystal properties. Insights unlocked in this study substantiate the correlations between the structure and function of these materials. These findings are invaluable in directing any future search for molecular crystal actuators of exceptional performance. The results also call for large-scale collaborations that expand on our collected dataset of dynamic crystals to improve its statistical significance and enhance the accuracy of the prediction model.

## Supporting Information Available

Experimental methods that elaborate on the data collection, visualization, and access to the code used to conduct the statistical analysis and build the machine learning model; definitions of actuation performance indices and crystal features, a summary of the data, comprehensive *t*-test results for the entire dataset, and the complete feature ranking results of the machine learning model; and ensemble learning training and validation loss curves (PDF)
Complete dataset including dynamic crystals (ZIP)

## References

[ref1] Swanson D. R. Undiscovered Public Knowledge. Libr. Q. 1986, 56, 103–118. 10.1086/601720.

[ref2] Takamizawa S.; Takasaki Y.; Sasaki T.; Ozaki N. Superplasticity in an Organic Crystal. Nat. Commun. 2018, 9, 3984 10.1038/s41467-018-06431-7. 30266968 PMC6162311

[ref3] Dharmarwardana M.; Welch R. P.; Kwon S.; Nguyen V. K.; McCandless G. T.; Omary M. A.; Gassensmith J. J. Thermo-Mechanically Responsive Crystalline Organic Cantilever. Chem. Commun. 2017, 53, 9890–9893. 10.1039/C7CC04346E. 28828435

[ref4] Zheng Y.; Jia X.; Li K.; Xu J.; Bu X.-H. Energy Conversion in Single-Crystal-to-Single-Crystal Phase Transition Materials. Adv. Energy Mater. 2022, 12, 2100324 10.1002/aenm.202100324.

[ref5] Dharmarwardana M.; Pakhira S.; Welch R. P.; Caicedo-Narvaez C.; Luzuriaga M. A.; Arimilli B. S.; McCandless G. T.; Fahimi B.; Mendoza-Cortes J. L.; Gassensmith J. J. Rapidly Reversible Organic Crystalline Switch for Conversion of Heat into Mechanical Energy. J. Am. Chem. Soc. 2021, 143, 5951–5957. 10.1021/jacs.1c01549. 33822596

[ref6] Huang Y.; Gong Q.; Yu J. Organic Crystal-Based Flexible Smart Materials. Sci. China Mater. 2022, 65, 1994–2016. 10.1007/s40843-021-1989-8.

[ref7] Wang C.; Sun C. C. The Landscape of Mechanical Properties of Molecular Crystals. CrystEngComm 2020, 22, 1149–1153. 10.1039/C9CE01874C.

[ref8] Koshima H. Mechanically Responsive Materials for Soft Robotics; John Wiley & Sons, 2020.

[ref9] Pons J. L. Emerging actuator technologies: a micromechatronic approach; John Wiley & Sons, 2005.

[ref10] Kitagawa D.; Tanaka R.; Kobatake S. Dependence of Photoinduced Bending Behavior of Diarylethene Crystals on Irradiation Wavelength of Ultraviolet Light. Phys. Chem. Chem. Phys. 2015, 17, 27300–27305. 10.1039/C5CP03073K. 26247682

[ref11] Hirano A.; Hashimoto T.; Kitagawa D.; Kono K.; Kobatake S. Dependence of Photoinduced Bending Behavior of Diarylethene Crystals on Ultraviolet Irradiation Power. Cryst. Growth Des. 2017, 17, 4819–4825. 10.1021/acs.cgd.7b00755.

[ref12] Kim T.; Zhu L.; Mueller L. J.; Bardeen C. J. Mechanism of Photoinduced Bending and Twisting in Crystalline Microneedles and Microribbons Composed of 9-Methylanthracene. J. Am. Chem. Soc. 2014, 136, 6617–6625. 10.1021/ja412216z. 24724968

[ref13] Tong F.; Xu W.; Guo T.; Lui B. F.; Hayward R. C.; Palffy-Muhoray P.; Al-Kaysi R. O.; Bardeen C. J. Photomechanical Molecular Crystals and Nanowire Assemblies Based on the [2+2] Photodimerization of a Phenylbutadiene Derivative. J. Mater. Chem. C 2020, 8, 5036–5044. 10.1039/C9TC06946A.

[ref14] Liu J.; Ye K.; Shen Y.; Peng J.; Sun J.; Lu R. Photoactuators Based on the Dynamic Molecular Crystals of Naphthalene Acrylic Acids Driven by Stereospecific [2+2] Cycloaddition Reactions. J. Mater. Chem. C 2020, 8, 3165–3175. 10.1039/C9TC06689F.

[ref15] Kitagawa D.; Tsujioka H.; Tong F.; Dong X.; Bardeen C. J.; Kobatake S. Control of Photomechanical Crystal Twisting by Illumination Direction. J. Am. Chem. Soc. 2018, 140, 4208–4212. 10.1021/jacs.7b13605. 29451385

[ref16] Zhang Z.; Mansouri Tehrani A.; Oliynyk A. O.; Day B.; Brgoch J. Finding the Next Superhard Material through Ensemble Learning. Adv. Mater. 2021, 33, 2005112 10.1002/adma.202005112. 33274804

[ref17] Peng J.; Xing J.; Bai J.; Ren Y.; Wang T.; Jia J. Spatial Photocontrol of the Passive Optical Output Direction of the Elastic Molecular Crystals Based on Acylhydrazone Derivatives. Dyes Pigm. 2021, 194, 109529 10.1016/j.dyepig.2021.109529.

[ref18] Gupta P.; Panda T.; Allu S.; Borah S.; Baishya A.; Gunnam A.; Nangia A.; Naumov P.; Nath N. K. Crystalline Acylhydrazone Photoswitches with Multiple Mechanical Responses. Cryst. Growth Des. 2019, 19, 3039–3044. 10.1021/acs.cgd.8b01860.

[ref19] Bushuyev O. S.; Corkery T. C.; Barrett C. J.; Friščić T. Photo-Mechanical Azobenzene Cocrystals and in Situ X-Ray Diffraction Monitoring of Their Optically-Induced Crystal-to-Crystal Isomerisation. Chem. Sci. 2014, 5, 3158–3164. 10.1039/C4SC00987H.

[ref20] Koshima H.; Ojima N.; Uchimoto H. Mechanical Motion of Azobenzene Crystals upon Photoirradiation. J. Am. Chem. Soc. 2009, 131, 6890–6891. 10.1021/ja8098596. 19453188

[ref21] Koshima H.; Ojima N. Photomechanical Bending of 4-Aminoazobenzene Crystals. Dyes Pigm. 2012, 92, 798–801. 10.1016/j.dyepig.2011.05.003.

[ref22] Zhu L.; Al-Kaysi R. O.; Bardeen C. J. Reversible Photoinduced Twisting of Molecular Crystal Microribbons. J. Am. Chem. Soc. 2011, 133, 12569–12575. 10.1021/ja201925p. 21749071

[ref23] Kim T.; Zhu L.; Mueller L. J.; Bardeen C. J. Dependence of the Solid-State Photomechanical Response of 4-Chlorocinnamic Acid on Crystal Shape and Size. CrystEngComm 2012, 14, 7792–7799. 10.1039/C2CE25811K.

[ref24] Zhu L.; Al-Kaysi R. O.; Bardeen C. J. Photoinduced Ratchet-Like Rotational Motion of Branched Molecular Crystals. Angew. Chem., Int. Ed. 2016, 55, 7073–7076. 10.1002/anie.201511444. 27150819

[ref25] Ohshima S.; Morimoto M.; Irie M. Light-Driven Bending of Diarylethene Mixed Crystals. Chem. Sci. 2015, 6, 5746–5752. 10.1039/C5SC01994J. 28757955 PMC5515063

[ref26] Nakagawa Y.; Morimoto M.; Yasuda N.; Hyodo K.; Yokojima S.; Nakamura S.; Uchida K. Photosalient Effect of Diarylethene Crystals of Thiazoyl and Thienyl Derivatives. Chem. – Eur. J. 2019, 25, 7874–7880. 10.1002/chem.201900811. 30934138

[ref27] Fujimoto A.; Fujinaga N.; Nishimura R.; Hatano E.; Kono L.; Nagai A.; Sekine A.; Hattori Y.; Kojima Y.; Yasuda N.; Morimoto M.; Yokojima S.; Nakamura S.; Feringa B. L.; Uchida K. Photoinduced Swing of a Diarylethene Thin Broad Sword Shaped Crystal: A Study on the Detailed Mechanism. Chem. Sci. 2020, 11, 12307–12315. 10.1039/D0SC05388K. 34094438 PMC8162954

[ref28] Park S. K.; Diao Y. Martensitic Transition in Molecular Crystals for Dynamic Functional Materials. Chem. Soc. Rev. 2020, 49, 8287–8314. 10.1039/D0CS00638F. 33021272

[ref29] Taniguchi T.; Fujisawa J.; Shiro M.; Koshima H.; Asahi T. Mechanical Motion of Chiral Azobenzene Crystals with Twisting upon Photoirradiation. Chem. – Eur. J. 2016, 22, 7950–7958. 10.1002/chem.201505149. 27097760

[ref30] Taniguchi T.; Asahi T.; Koshima H. Photomechanical Azobenzene Crystals. Crystals 2019, 9, 437 10.3390/cryst9090437.

[ref31] Zhou B.; Yan D. Recent Advances of Dynamic Molecular Crystals with Light-Triggered Macro-Movements. Appl. Phys. Rev. 2021, 8, 041310 10.1063/5.0059919.

[ref32] Huang C.; Huang R.; Zhang S.; Sun H.; Wang H.; Du B.; Xiao Y.; Yu T.; Huang W. Recent Development of Photodeformable Crystals: From Materials to Mechanisms. Research 2021, 2021, 1–26. 10.34133/2021/9816535. PMC8605404 34870227

[ref33] Rath B. B.; Vittal J. J. Single-Crystal-to-Single-Crystal [2 + 2] Photocycloaddition Reaction in a Photosalient One-Dimensional Coordination Polymer of Pb(II). J. Am. Chem. Soc. 2020, 142, 20117–20123. 10.1021/jacs.0c09577. 33175523

[ref34] Seki T.; Mashimo T.; Ito H. Anisotropic Strain Release in a Thermosalient Crystal: Correlation between the Microscopic Orientation of Molecular Rearrangements and the Macroscopic Mechanical Motion. Chem. Sci. 2019, 10, 4185–4191. 10.1039/C8SC05563G. 31057747 PMC6471989

[ref35] Kitagawa D.; Okuyama T.; Tanaka R.; Kobatake S. Photoinduced Rapid and Explosive Fragmentation of Diarylethene Crystals Having Urethane Bonding. Chem. Mater. 2016, 28, 4889–4892. 10.1021/acs.chemmater.6b02017.

[ref36] Natekin A.; Knoll A. Gradient Boosting Machines, a Tutorial. Front. Neurorobot. 2013, 7, 21 10.3389/fnbot.2013.00021. 24409142 PMC3885826

[ref37] Friedman J. H. Greedy Function Approximation: A Gradient Boosting Machine. Ann. Stat. 2001, 29, 1189–1232. 10.1214/aos/1013203451.

[ref38] Koshima H.; Taniguchi T.; Asahi T. Mechanically Responsive Crystals by Light and Heat. In Mechanically Responsive Materials for Soft Robotics, Koshima H., Ed.; Wiley, 2020; pp. 57–82. 10.1002/9783527822201.ch3

[ref39] Michel F.; Ehrfeld W. Mechatronic micro devices. In MHS’99: Proceedings of 1999 international symposium on micromechatronics and human science, Institute of Electrical and Electronics Engineers: Nagoya Japan, November 23–26, , 1999; pp. 27–34. 10.1109/MHS.1999.819978

