# Supplementary material for: Intersectional Effects of Crystal Features on the Actuation Performance of Dynamic Molecular Crystals
Source: J Am Chem Soc. 2023 May 26;145(22):12173–80. doi: 10.1021/jacs.3c02184 (PMC10251518; doi:10.1021/jacs.3c02184)
Supplement: Supplementary file 1 — ja3c02184_si_001.pdf [file ja3c02184_si_001.pdf]

Supporting Information

Intersectional Effects of Crystal Features on the Actuation Performance of Dynamic Molecular Crystals

Jad Mahmoud Halabi,[1] Marieh B. Al-Handawi,[1] Rodrigo Ceballos,[2] Pance Naumov[1,3,4,5]*

[1]*Smart Materials Lab, New York University Abu Dhabi, PO Box 129188, Abu Dhabi, UAE*

[2]*Paige.ai, New York City, NY 10036, USA*

[3]*Center for Smart Engineering Materials, New York University Abu Dhabi, PO Box 129188, Abu Dhabi, UAE*

[4]*Research Center for Environment and Materials, Macedonian Academy of Sciences and Arts, Bul. Krste Misirkov 2, MK–1000 Skopje, Macedonia*

[5]*Molecular Design Institute, Department of Chemistry, New York University, 100 Washington Square East, New York, NY 10003, USA*

This PDF Includes:

I. Experimental Methods
II. Tables S1 to S7
III Figure S1

I. SUPPORTING EXPERIMENTAL METHODS

Data collection: The data utilized in this study have been primarily sourced from published literature between 2006 and 2020 with 68 unique studies, encompassing a range of actuation performance metrics and various crystal features for molecular crystals. In instances where values on the actuation performance were not explicitly stated in the literature, we employed editing software to extract information such as the dimensions of crystals or response and recovery times of the actuation, enabling us to calculate the additional properties and better populate the dataset. In instances where physical, chemical, or mechanical properties were not explicitly specified in the same literature, we resorted to publicly available datasets or alternative literature that report on the same molecule or single crystal. X-ray diffraction information was obtained from the Cambridge Structural Database (CSD) on the same single crystal systems. Where available, we used the crystallography information from the same study that reports on actuation performance. Given that actuation performance studies on molecular single crystals do not always conduct X-ray different analyses, we used the most recently published data that more closely matches the experimental conditions followed during actuation.

We have made every effort to ensure the accuracy of the data collected and the integrity of our analysis. However, it is important to note that we did not conduct any additional experimentation or validation beyond the information provided in the literature. We rely on the accuracy of the original published data, and as such, cannot be held responsible for any discrepancies or inaccuracies that may arise from the source material. By using the data and findings presented in this paper, the reader acknowledges and agrees that the authors are not liable for any errors, discrepancies, or inaccuracies in the original published data. In case any such discrepancies arise or in case the reader has any questions or concerns, please reach out directly to the corresponding author.

We recognize the value of continuous improvement and expansion of the dataset used in this study. As such, we invite and encourage collaboration within the research community to build upon the current data set with new examples of tested and characterized molecular crystals. By populating areas where data may be sparse, refining existing data points through re-experimentation, and incorporating new information, we can collectively enhance the learning capabilities and predictive accuracy of the machine learning model employed in this work.

Dynamic molecular crystals sample: The molecular crystals included in the dataset encompass a wide range of photoresponsive and thermoresponsive single crystals. While we recognize there are other examples of dynamic molecular crystals such mechanoresponsive, electroresponsive, and humidity-responsive crystals, these types of crystals are not as widely and extensively reported on and thereby are excluded from our dataset. Additionally, we recognize the variety of underlying chemical transformations and molecule types within photoresponsive (ex: t-type and p-type photochromic molecules) and thermoresponsive crystals. These differences are accounted for through different crystal features such as chemical families, reaction groups, and classification of single crystals by whether they undergo phase transition transformations, martensitic phase transition transformations, or not. Similarly, and due to few examples reported in the literature, crystals that undergo bending, contraction/expansion, and jumping represent the majority of the samples captured in the data. While the dataset does include crystals that undergo twisting, due to the small sample size, we excluded this type of deformation from the analysis.

Note: A molecular crystal actuation event defined in this work represents the introduction of a single stimulus at a time and a full actuation cycle starts with the introduction of a stimulus and ends after the crystal has gone back to its original condition or has reached steady state after which no motion can be recorded without the introduction of an additional stimulus.

Ashby Plots: The Ashby plots in this work were constructed using python and the dataset was collected through a wide literature review on microactuators and a range of soft to hard actuators reported on in recent studies. Additional examples of commercially available actuators were extracted from the ANSYS Granta Materials database.

Statistical Analysis and Machine Learning code: The code python files have been uploaded to a public github repository and can be accessed through this link: https://github.com/jmah13/Dynamic-Molecular-Crystals.

II. SUPPORTING TABLES

Supporting Table S1 | Definition of performance indices. A list of the performance indices relevant to actuator design along with the respective equations, and definitions

Performance Indices	Symbol / Unit	Equations	Definitions & Remarks
**Free stroke**	ΔL or $\delta_{\max}$ / m	Measured	*The maximum stroke or displacement the crystal can achieve under no external load. When not reported, this property was determined from scaled images and videos. For bending crystals, it was determined as the maximum tip displacement*
**Deformation capacity**	δ_{capacity} / unitless	$\delta_{\text{capacity}} = \frac{\delta_{\max}}{L_{\text{active}}}$	*The ratio of the free stroke over the length along the active axis of actuation. In case of axial expansion or contraction deformation capacity is equivalent to deformation strain*
**Response time**	t_{response} / s	Measured	*The time the crystal takes to reach maximum stroke. For photoresponsive crystals it is recorded starting when irradiation hits the crystal. For thermoresponsive crystals, it is recorded starting when the administered temperature reaches the activation temperature. Note that the rate of heating is likely to affect the response time but it is not accounted for here for the lack of necessary information about the experimental setup*
**Recovery time**	t_{recovery} / s	Measured	*The time the crystal takes to return to its original form and position if the actuation is fully reversible*
**Velocity**	v / m s^{-1}	$v = \frac{\delta_{\max}}{t_{\text{response}}}$	*The calculated maximum velocity of the unrestrained actuator*
**Force output**	F / N	Measured or calculated for object-lifting experiments as: $F = m.g$ m: mass of object g: gravity	*The force generated by the crystals is typically measured using micro-sensors, micro-fabricated devices, pushing/lifting objects of measured weight. This property does not always represent the maximum force and is often less than the blocking force*
**Force density**	F_{density} / N m^{-3}	$F_{\text{density}} = \frac{\mathbf{F}}{V}$ V: total volume	*The force output per unit volume calculated from the dimensions of the crystals*
**Work output**	W / J	$W = \mathbf{F}.\delta$	*Calculated from the force output and the displacement under loading. Note that when the force output is the blocking force, the work output cannot be determined since there is no displacement and the quantity is effectively null*
**Work capacity**	W_{capacity} / J m^{-3}	$W_{\text{capacity}} = \frac{W}{V}$	*The volumetric work density where the volume is calculated from the dimensions of the crystals*
**Power output**	P / kW	$P = \frac{W}{t}$	*The work output per second calculated using the time needed to displace a load by δ*

**Power density**	P_{density} / kW m^{-3}	$P_{\text{density}} = \frac{P}{V}$	*The volumetric power density where the volume is calculated from the dimensions of the crystals*
**Efficiency**	η / unitless	<u>Photoactuation efficiency</u><br>$\eta_{\text{photo}} = \frac{P}{P_{\text{irrad}} \cdot S_{\text{irrad}}}$<br>$P_{\text{irrad}}$: irradiation power per unit area (kW m^{-2})<br>S_{irrad}: irradiated surface area (m^2)<br><br><u>Thermoactuation efficiency</u><br>$\eta_{\text{thermo}} = \frac{W}{m c_{\text{p}} \Delta T}$<br>$m$: mass of sample<br>$c_{\text{p}}$: specific heat capacity<br>ΔT: change in temperature	*The ratio of the output mechanical power over the thermal or irradiation power.*<br>*Note that because the specific heat capacity was often not reported for these crystals, the efficiency of many thermoresponsive crystals is not reported*

Supporting Table S2 | Actuation performance data. A list of all reported static and dynamic performance indices of molecular crystals' actuation behavior. The spread of data and sample size for each index are reported

	**Performance Indices**	**Count**	**Mean**	**Std**	**Min**	**25%**	**50%**	**75%**	**Max**
STATIC PERFORMANCE	Free stroke / m	94	$2.21\text{x}10^{-3}$	$1.07\text{x}10^{-2}$	$2.00\text{x}10^{-7}$	$1.75\text{x}10^{-5}$	$8.55\text{x}10^{-5}$	$3.17\text{x}10^{-4}$	$9.00\text{x}10^{-2}$
	Deformation capacity	93	5.20	44.5	$1.49\text{x}10^{-3}$	$7.14\text{x}10^{-2}$	0.197	0.495	$4.29\text{x}10^{2}$
	Force output / N	28	$1.05\text{x}10^{-2}$	$2.94\text{x}10^{-2}$	$1.25\text{x}10^{-8}$	$9.60\text{x}10^{-5}$	$3.78\text{x}10^{-4}$	$1.40\text{x}10^{-3}$	0.132
	Force density / N m^{-3}	26	$3.42\text{x}10^{9}$	$1.62\text{x}10^{10}$	$3.70\text{x}10^{2}$	$2.72\text{x}10^{6}$	$6.46\text{x}10^{7}$	$3.12\text{x}10^{8}$	$8.29\text{x}10^{10}$
	Response time / s	101	14.9	47.0	$2.00\text{x}10^{-4}$	$3.50\text{x}10^{-2}$	1.50	6.00	$3.00\text{x}10^{2}$
	Recovery time / s	38	54.4	$1.06\text{x}10^{2}$	$2.00\text{x}10^{-3}$	1.00	11.5	49.0	$4.80\text{x}10^{2}$
	Velocity / m s^{-1}	91	0.15	0.58	$2.10\text{x}10^{-8}$	$2.21\text{x}10^{-5}$	$3.13\text{x}10^{-4}$	$4.10\text{x}10^{-3}$	4.70
DYNAMIC PERFORMANCE	Work output / J	44	$1.06\text{x}10^{-5}$	$6.78\text{x}10^{-5}$	$3.75\text{x}10^{-13}$	$1.51\text{x}10^{-10}$	$3.67\text{x}10^{-9}$	$6.62\text{x}10^{-8}$	$4.50\text{x}10^{-4}$
	Work capacity / J m^{-3}	42	$7.12\text{x}10^{3}$	$1.65\text{x}10^{4}$	$5.00\text{x}10^{-5}$	21.7	$6.52\text{x}10^{2}$	$2.69\text{x}10^{3}$	$8.13\text{x}10^{4}$
	Power output / kW	35	$2.33\text{x}10^{-6}$	$1.37\text{x}10^{-5}$	$3.33\text{x}10^{-16}$	$1.90\text{x}10^{-12}$	$2.96\text{x}10^{-11}$	$7.13\text{x}10^{-10}$	$8.10\text{x}10^{-5}$
	Power density / kW m^{-3}	35	$8.74\text{x}10^{2}$	$2.90\text{x}10^{3}$	$5.14\text{x}10^{-4}$	0.487	2.77	84.0	$1.53\text{x}10^{4}$
	Efficiency / η	23	$1.50\text{x}10^{-4}$	$5.49\text{x}10^{-4}$	$3.53\text{x}10^{-10}$	$1.97\text{x}10^{-7}$	$1.00\text{x}10^{-6}$	$3.37\text{x}10^{-5}$	$2.65\text{x}10^{-3}$

Supporting Table S3 | Categorical features. A list of all available categorical data of molecular crystals that explains their chemical nature, reactivity, and responsive behavior

Categorical Features	Explanation	Categories	
**Chemical family**	*General chemical families each entry belong to*	- Acids<br>- Amphiphilic crystals<br>- Anthracenes (AC)<br>- Arylidene-furanones<br>- Arylidene-imidazolones<br>- Azobenzenes (AZ)<br>- Benzoxazoles<br>- Co-crystals<br>- Diarylethenes (DE)<br>- Halogenated benzenes<br>- Hydrazides<br>- Fluorenones	- Furylfulgides<br>- Methylbenzenes<br>- Organometallic<br>- Olefin pairs<br>- Organic salts<br>- Perylenes<br>- Peptides<br>- Perovskites<br>- Pincer-type diesters<br>- Naphthalene diimides<br>- Sesquiterpene lactones<br>- Salicylidenes
**Reaction group**	*The characteristic type of chemical reaction driving the response*	- Ring-opening isomerization<br>- Photodimerization<br>- Configurational Isomerization (trans-cis/ E-Z, keto-enol)<br>- Molecular gliding<br>- Molecular rotation	- Conformational change<br>- Molecular gliding/conformational<br>- Molecular gliding/ rotation
**Martensitic reaction**	*Diffusionless versus non-diffusionless phase transitions*	- Yes	- No
**Phase transition**	*Phase transition crystal transformations*	- Yes	- No
**Organo-metallic**	*Presence of an organometallic complex*	- Yes	- No
**Type of Deformation**	*Depends on SCSC vs surface transformations*	- Bending<br>- Twisting<br>- Jumping	- Axial expansion/ contraction
**Source of actuation**	*Type of stimulus driving the response*	- Photo	- Thermo
**Crystal habit**	*Geometric appearance of the crystal*	- Blade<br>- Prism<br>- Tabular<br>- Parallelepiped	- Plate<br>- Needle<br>- Block<br>- Rod
**Crystal family**	*Crystal systems based on unit cell*	- Triclinic<br>- Orthorhombic	- Hexagonal<br>- Monoclinic
**Reversibility**	*The crystal is able to retain its original state once stimulus is removed*	- Yes	- No
**Crystal intact**	*Structural integrity of crystal after actuation is preserved*	- Yes	- No

Supporting Table S4 | Continuous features. A list of all available continuous features of molecular crystals including dimensions and physical properties, crystal lattice parameters, and molecular information. The spread of data and sample size for each feature are reported

	Continuous Features	Count	Mean	Std	Min	25%	50%	75%	Max
PHYSICAL & MECHANICAL PROPERTIES	Length / m	106	2.11x10^{-3}	6.50x10^{-3}	6.00x10^{-6}	2.03x10^{-4}	5.15x10^{-4}	1.53x10^{-3}	6.00x10^{-2}
	Width / m	83	2.62x10^{-4}	5.11x10^{-4}	1.77x10^{-7}	3.60x10^{-5}	1.10x10^{-4}	2.41x10^{-4}	3.75x10^{-3}
	Thickness / m	89	1.07x10^{-4}	2.63x10^{-4}	1.77x10^{-7}	6.00x10^{-6}	1.90x10^{-5}	5.15x10^{-5}	2.00x10^{-3}
	Volume / m^3	85	6.94x10^{-10}	3.70x10^{-9}	2.35x10^{-19}	2.38x10^{-13}	2.64x10^{-12}	3.10x10^{-11}	3.00x10^{-8}
	Weight / kg	34	1.92x10^{-7}	4.71x10^{-7}	1.28x10^{-11}	2.83x10^{-9}	1.29x10^{-8}	9.00x10^{-8}	2.27x10^{-6}
	E / GPa	17	5.87	4.16	0.760	2.60	3.80	8.50	1.50x10^{1}
	Melting point / °C	40	1.51x10^{2}	43.7	78.0	1.24x10^{2}	1.57x10^{2}	1.71x10^{2}	2.51x10^{2}
	Density / kg m^{-3}	113	1.45	0.26	0.91	1.32	1.42	1.52	3.02
CRYSTAL LATTICE PARAMETERS	Z Value	116	4.00	2.53	1.00	2.00	4.00	4.00	18.0
	a	117	11.7	9.26	3.77	7.12	9.77	12.4	59.4
	b	117	12.2	6.43	3.77	7.27	11.2	13.8	33.8
	c	117	19.2	9.46	4.02	12.6	17.4	23.1	60.1
	Alpha	116	89.2	6.53	70.7	89.4	90.0	90.0	1.09x10^{2}
	Beta	116	93.5	9.45	73.6	89.6	93.1	99.5	1.24x10^{2}
	Gamma	116	90.3	6.22	62.4	90.0	90.0	90.0	1.20x10^{2}
	Cell volume	117	2.19x10^{3}	1.50x10^{3}	1.69x10^{2}	1.17x10^{3}	2.06x10^{3}	2.46x10^{3}	1.09x10^{4}
	Unique chemical units	106	1.49	0.819	1.00	1.00	1.00	2.00	4.00
	Molecular volume	104	6.17x10^{2}	3.34x10^{2}	1.47x10^{2}	3.58x10^{2}	5.74x10^{2}	7.85x10^{2}	1.70x10^{3}
	Molecular weight / g mol^{-1}	117	5.19x10^{2}	2.66x10^{2}	1.29x10^{2}	2.90x10^{2}	4.95x10^{2}	6.83x10^{2}	1.22x10^{3}

Supporting Table S5 | Two-sample t-test results. All the t-tests of significant difference between any pair of categories with respect to continuous performance features are shown. The minimum significant sample size for each category was set at 10 and the confidence interval at 95%

Categorical Feature	Category 1 Name	Category 2 Name	Cat. 1 Size	Cat. 2 Size	Performance Index	p-value
**Chemical family**	Acids	Organometallic	10	19	Free stroke	3.77×10^{-3}
	DE	Organometallic	26	19	Free stroke	6.86×10^{-5}
	Acid	Organometallic	10	18	Deformation capacity	1.18×10^{-2}
	DE	Organometallic	26	18	Deformation capacity	1.37×10^{-3}
	Acid	Organometallic	10	24	Response time	1.71×10^{-2}
	DE	Organometallic	20	24	Response time	3.45×10^{-3}
**Reaction group**	Molecular gliding	Photodimerization	10	11	Free stroke	3.18×10^{-2}
	Molecular rotation	Photodimerization	14	11	Free stroke	3.53×10^{-4}
	Molecular rotation	Ring-opening	14	27	Free stroke	1.00×10^{-5}
	Photodimerization	Ring-opening	11	27	Free stroke	3.32×10^{-2}
	Isomerization	Ring-opening	17	27	Deformation capacity	3.64×10^{-2}
	Molecular gliding	Molecular rotation	10	14	Deformation capacity	2.49×10^{-2}
	Molecular rotation	Photodimerization	14	11	Deformation capacity	2.11×10^{-3}
	Molecular rotation	Ring-opening	14	27	Deformation capacity	1.09×10^{-3}
	Isomerization	Photodimerization	15	21	Velocity	1.54×10^{-3}
	Isomerization	Ring-opening	15	20	Velocity	3.91×10^{-2}
	Molecular gliding	Photodimerization	11	21	Velocity	2.19×10^{-3}
	Molecular gliding	Ring-opening	11	20	Velocity	2.32×10^{-2}
	Molecular rotation	Photodimerization	10	21	Velocity	4.93×10^{-2}
	Isomerization	Molecular rotation	17	12	Response time	2.83×10^{-3}
	Molecular gliding	Molecular rotation	13	12	Response time	1.82×10^{-2}
	Molecular rotation	Ring-opening	12	21	Response time	2.69×10^{-3}
**Martensitic transition**	No	Yes	64	15	Deformation capacity	2.19×10^{-2}
	No	Yes	28	12	Work capacity	2.76×10^{-4}
	No	Yes	20	11	Power output	3.06×10^{4}
	No	Yes	68	21	Response time	2.77×10^{-3}
**Phase transition**	No	Yes	53	41	Free stroke	4.75×10^{-3}
	No	Yes	53	40	Deformation capacity	2.09×10^{-5}
	No	Yes	44	47	Velocity	1.04×10^{-3}
	No	Yes	12	16	Force output	3.48×10^{-2}
	No	Yes	12	23	Power output	1.64×10^{-3}
	No	Yes	49	52	Response time	6.18×10^{-10}
	No	Yes	20	18	Recovery time	1.19×10^{-3}
	No	Yes	10	13	Efficiency	2.56×10^{-3}

**Source of actuation**	Photo	Thermo	61	29	Deformation capacity	5.77x10^{-4}
	Photo	Thermo	16	11	Force output	1.00x10^{-2}
	Photo	Thermo	29	14	Work capacity	5.29x10^{-5}
	Photo	Thermo	21	13	Power output	2.09x10^{-5}
	Photo	Thermo	64	34	Response time	1.11x10^{-3}
	Photo	Thermo	25	11	Recovery time	3.28x10^{-3}
**Type of deformation**	Bending	Jumping	52	12	Free stroke	1.47x10^{-3}
	Bending	Expansion/contraction	52	27	Free stroke	2.86x10^{-9}
	Bending	Jumping	51	12	Deformation capacity	7.40x10^{-5}
	Bending	Expansion/contraction	51	27	Deformation capacity	1.60x10^{-5}
	Bending	Jumping	45	24	Velocity	1.92x10^{-13}
	Bending	Expansion/contraction	45	19	Velocity	3.27x10^{-2}
	Jumping	Expansion/contraction	24	19	Velocity	2.17x10^{-10}
	Bending	Expansion/contraction	11	11	Force output	2.61x10^{-2}
	Bending	Jumping	47	30	Response time	1.06x10^{-10}
	Jumping	Expansion/contraction	30	20	Response time	1.55x10^{-3}
	Bending	Expansion/contraction	23	11	Recovery time	1.71x10^{-2}

Supporting Table S6 | Ensemble learning feature importance scores. A list of features with associated feature importance scores (F_{score}) normalized for each actuation performance index demonstrating each feature's contribution to the learning model

Free Stroke		Force Output		Response Time		Velocity	
**F_{score}**	**Feature**	**F_{score}**	**Feature**	**F_{score}**	**Feature**	**F_{score}**	**Feature**
0.2169	Type of deformation	0.1883	Width	0.1346	Type of deformation	0.3582	Type of deformation
0.2085	Length	0.1014	Thickness	0.0969	Martensitic	0.0617	Density
0.1224	Molecular volume	0.0983	Molecular volume	0.0706	Cell volume	0.0448	Source of actuation
0.0585	Unique chem. units	0.0870	Weight	0.0646	b	0.0441	Weight
0.0491	Source of actuation	0.0790	Length	0.0636	Crystal family	0.0417	Chemical family
0.0367	Thickness	0.0616	Source of actuation	0.0620	Organometallic	0.0415	Length
0.0297	Organometallic	0.0461	*a*	0.0583	*a*	0.0371	Cell volume
0.0259	Crystal habit	0.0435	Molecular weight	0.0570	Phase transition	0.0296	Crystal family
0.0252	Molecular weight	0.0433	Type of deformation	0.0447	Reaction group	0.0280	*a*
0.0224	Density	0.0416	Organometallic	0.0410	Source of actuation	0.0275	Reaction group
0.0219	*c*	0.0329	Martensitic	0.0337	Crystal habit	0.0273	Reversibility
0.0188	Martensitic	0.0223	*b*	0.0288	Reversibility	0.0258	*b*
0.0175	Chemical family	0.0214	Cell volume	0.0272	Thickness	0.0249	Organometallic

Work Capacity		Power Output		Efficiency	
**F_{score}**	**Feature**	**F_{score}**	**Feature**	**F_{score}**	**Feature**
0.2164	Crystal habit	0.0983	Phase transition	0.2064	Martensitic
0.0849	Source of actuation	0.0942	Source of actuation	0.0849	Type of deformation
0.0814	Martensitic	0.0684	Reaction group	0.0667	*Beta*
0.0555	Crystal family	0.0647	Chemical family	0.0613	Phase transition
0.0518	Type of deformation	0.0560	Cell volume	0.0493	Chemical family
0.0471	Width	0.0528	Crystal habit	0.0426	*b*
0.0434	*c*	0.0517	Molecular volume	0.0383	Molecular weight
0.0418	Length	0.0391	*a*	0.0362	Crystal family
0.0394	*a*	0.0379	Melting point	0.0342	Density
0.0393	Reversibility	0.0368	Thickness	0.0325	*c*
0.0384	*Beta*	0.0351	Organometallic	0.0320	Width
0.0382	Organometallic	0.0340	*c*	0.0312	Molecular volume
0.0293	Reaction group	0.0313	Width	0.0288	Reaction group

Supporting Table S7 | Classes of actuators. All actuator classes and the sub-families within each class considered in the comparison with molecular crystals

Actuator Class	Types of Actuators	
Electromagnetic Devices	- Electric Cylinders<br>- Voice Coil Motors<br>- Solenoids<br>- Mini-Linear	
Magnetostriction	Ferromagnetic Actuators	
MEMS	Micro-Electromechanical Systems	
Fluidic	- Hydraulic<br>- Pneumatic	
Piezoelectric	- Ceramic Piezoelectric<br>- Electrostrictive Actuators	
Shape Memory Alloys	Shape Memory-NiTi	
Thermally Actuated	Bimetallic Strips	
Smart Materials	Nano-muscles	
Hydrogels	(single class)	
Responsive Polymers	Electroactive Polymers (EAP) – Ionic	- Ionic Polymer–Metal Composites (IPMC)<br>- Conductive Polymers (CP)<br>- Polymer Carbon Nanotubes (CNT)
	Electroactive Polymers (EAP) – Electronic	- Dielectric Elastomers (DEA)<br>- Liquid Crystal Elastomers (LCE)
	Shape Memory Polymers (SMP)	
Natural	Muscle	
Molecular Crystals	- Photo/Thermoresponsive<br>- Organic Single-crystal Solids	

III. SUPPORTING FIGURES

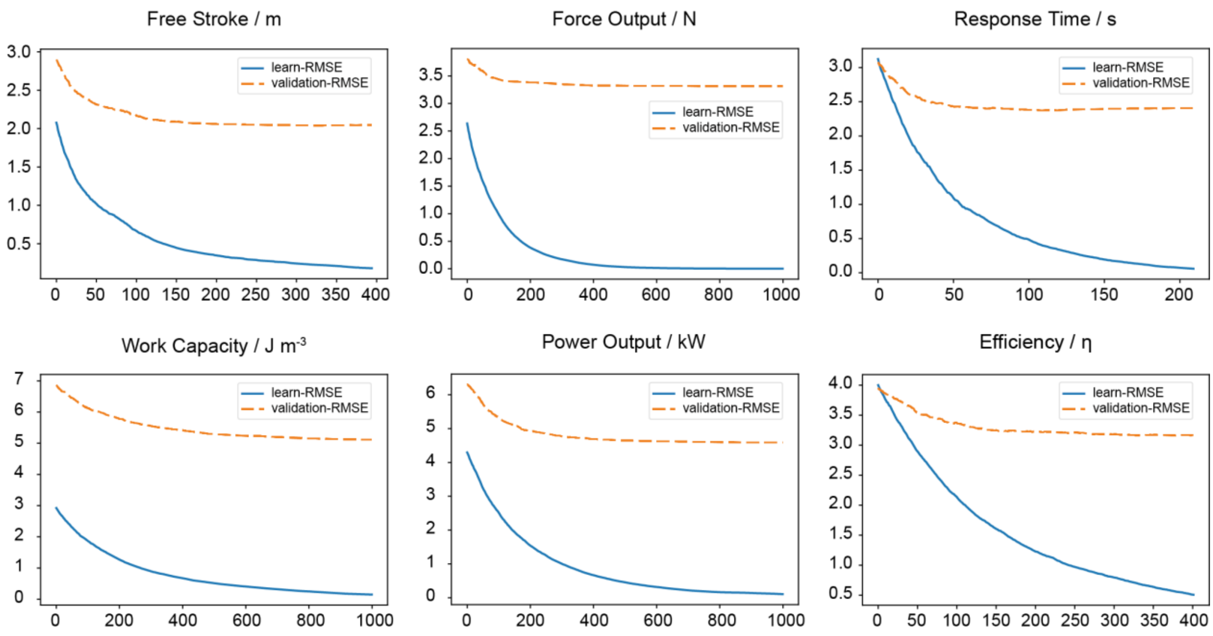

Supporting Figure S1 | Learning ensemble training and validation losses. Root mean squared error (RMSE) log-scale learning and validation loss functions to evaluate the performance of the machine learning model.
